# The Quality of Breakfast and Healthy Diet in School-aged Adolescents and Their Association with BMI, Weight Loss Diets and the Practice of Physical Activity

**DOI:** 10.3390/nu12082294

**Published:** 2020-07-30

**Authors:** Raquel M. Guevara, José D. Urchaga, Antonio S. Cabaco, José E. Moral-García

**Affiliations:** Faculty of Education, C/ Henry Collet, Pontifical University of Salamanca, 37007 Salamanca, Spain; rmguevarain@upsa.es (R.M.G.); asanchezca@upsa.es (A.S.C.); jemoralga@upsa.es (J.E.M.-G.)

**Keywords:** dietary habits/diet, breakfast, weight loss diet, BMI, physical activity, eating disorders

## Abstract

Dietary habits are an important factor in the protection of adolescent health. The quality and frequency of breakfast and the various food groups can affect the wellbeing of this population group in both the short and long term. Research indicates that there is a range of relevant variables in the study of diet at this stage: following a weight loss diet, body mass index and the practice of physical exercise, amongst others. In this paper, all three variables are analysed, together with others of a demographic nature (sex and age). This is a descriptive cross-sectional survey that was carried out on 1318 adolescents aged 11 to 18. The Health Behaviour in School-Aged Children (HBSC) international study questionnaire, sponsored by the World Health Organisation, was used. In general terms, the data revealed that the majority of adolescents do not have adequate eating patterns. The quality and frequency of breakfast and the consumption of food types are associated with almost all the variables under study; additionally, there are significant differences by sex and school year. Finally, proposals are made to prevent eating disorders, which are appearing at an increasingly young age.

## 1. Introduction

Our diet, together with physical activity (PA), are the main factors contributing to the protection of our health in all stages of life. Therefore, adopting and maintaining healthy habits in both spheres provides numerous benefits and contributes to the maintenance or improvement of our quality of life [1,2]. During adolescence, the adoption of adequate dietary and exercise habits can protect against obesity and prevent chronic diseases, such as diabetes mellitus, heart disease or stroke [3,4].

In Spain, overweight and obesity affect 34.4% of the young population between 5 and 19 years of age [5], making it a public health priority on account of the consequences this may have in the later stages of life [6,7].

The World Health Organisation’s (WHO) dietary recommendations for adolescents [2] indicate that their diet must be varied and rich in nutrients. Additionally, international agencies think it is relevant to take into account the weekly and/or daily frequency with which the various food groups need to be consumed in order to achieve a balanced diet. Recent research studies undertaken by experts in the field indicate that the number of young people who have eating disorders or unbalanced eating habits is high [8,9]. The WHO [10] and various international agencies recommend for three or more pieces of fruit and vegetables to be eaten daily, the rotation between white meats and fish, and only an occasional consumption of red meats, snacks, confectionery and soft drinks. Research studies [11,12,13] highlight that the percentage of young people who maintain eating patterns that are not consistent with a healthy diet is high (low consumption of fruit and vegetables, frequent consumption of snacks, confectionery and soft drinks, reduction in the consumption of dairy products as they grow up, etc.). The ENALIA research, carried out by the Spanish Agency for Consumption, Food Safety and Nutrition, points out that less than half of Spanish adolescents follow the nutritional recommendations [14]. Only 18.2% of the young population consume fruit with optimal frequency (every day, more than once), whereas 13.6% consume vegetables; 26.2% consume confectionery within the recommended frequency (occasionally, less than once a week) and 34.5% of adolescents have soft drinks occasionally (according to recommendations) [15]. There are differences in the consumption levels of some foods by sex: girls eat fruit and vegetables more often, but also more confectionery, whereas boys have soft drinks more often [15]. By age group, the consumption of fruit and vegetables compared with the optimal level decreases as age increases and, whereas the consumption of confectionery and snacks remains the same with age, the consumption of soft drinks increases. The association between the food adolescents eat and the body mass index (BMI) is clear, with those individuals with the best dietary habits showing lower BMIs [11,16]. Following a weight loss diet affects and limits the consumption of certain types of food, frequently removing fats, confectionery, fried food and sweetened drinks from the diet. In this sense, following a specific weight loss diet tends to involve the consumption of natural and healthy food, such as fruit and vegetables [17], although at this stage of development diets are mostly associated with an obsession with thinness or a sense of dissatisfaction with one’s own body, which leads adolescents to follow such diets without medical supervision. Food restrictions or skipping meals without adequate supervision can lead to negative health consequences for young people and cause eating disorders such as anorexia, bulimia, etc. [18,19]. Given that adolescence is a period of time where many changes occur, it is relevant for young boys and girls to learn to accept their bodies [20]. However, when young people do not adhere to adequate eating patterns, their physical self-concept decreases, especially in girls, which in turn derives relational or academic difficulties [20]. Other studies, for example, state that skipping meals in adolescence affects the ovarian and uterine functions in girls [21]. In Spain, 13.1% of adolescents state that they are on some type of weight loss diet or other, although this is more frequent in girls and at younger ages [15]. Physical activity also affects the dietary habits of adolescents [22,23], with boys who eat better practicing more physical activity.

Breakfast is the first meal of the day and breaks up the long period of fasting taking place while we sleep. It is consumed within 2 to 3 h from waking up. It is made of food and drinks from at least one food group and can be eaten anywhere [24]. This meal helps reorganise the metabolic changes taking place at night time, prevents the effects of a long fasting period and, on many occasions, it includes the first liquid that is consumed after a long period of time, which means that it contributes to maintaining adequate water levels [25]. The quality and frequency of breakfast is also very relevant when considering a healthy diet. In spite of the fact that it is currently necessary to reach more solid scientific consensus in terms of the food that must be included in breakfast [26,27], at least three of the following main food groups are proposed as essential: dairy products, cereal and fruit, which need to be consumed every day of the week [28]. The breakfast that is most frequently consumed in Spain consists of a dairy product—specifically skimmed or semi-skimmed milk and a cereal—especially bread, which may differ from other European countries [29,30,31,32]. Research shows that this type of breakfast is a good indicator of a healthy lifestyle [33], associating adherence to it with a better quality diet [27,34]. There is plenty of research that indicates this is essential in the prevention of disease. In Huasan’s study, for example, this is considered to be an essential practice to prevent type II diabetes [35]. On the other hand, skipping breakfast has been associated with poor-quality diets, lower cognitive performance and various other negative impacts on health [26,36]. In spite of the importance of breakfast, skipping breakfast or having a poor-quality breakfast is frequent in adolescents [37]. Research such as that of Monzani or Wadoloska associate skipping breakfast with general and central obesity [38,39]. In girls it is also associated with more frequent dysmenorrhea and irregular menstruation [21]. In Spain, the percentage of high school boys going to class without having had breakfast is 16.9% [15]. If we take into account social-demographic variables such as sex, the national data indicate that girls tend to skip breakfast more frequently, with the boys having healthier habits in this regard. Age is also influential on the habit of eating breakfast, with older adolescents’ habits being worse. Thus, the percentage of high school boys going to class without having had any breakfast at all increases from 8.2% at 11–12 years of age to 24.6% at 17–18 years of age [15]. Numerous studies point out that regular consumption of breakfast has also been associated with lower BMI [40,41,42,43]. Adherence to a weight loss diet can lead to skipping breakfast altogether or having an unhealthy breakfast, mainly as a result of wanting to stay in shape or lose weight, with this habit being more frequent amongst girls [44]. Lastly, research reveals that young people skipping breakfast tend to have a higher BMI and tend to practice less physical activity [45,46,47].

In this context, this study intends to analyse the range of variables previously mentioned (i.e., sex, age, weight loss diet, BMI and PA) and their effect on the dietary habits of adolescents. The specific objectives of this study are to: (a) understand the quality of breakfast and the diet of adolescents by sex, school year, weight loss diet adherence, BMI and the practice of physical activity; (b) analyse how frequently they tend to have breakfast (on weekdays, on the weekend and for the entire week) and the various food groups during the other daily meals by sex, school year, diet adherence, BMI and PA that adolescents have; and (c) understand which of the variables (sex, school year, diet adherence, BMI and physical activity) is the most influential in predicting breakfast habits and the daily or occasional consumption of food groups during the other daily meals.

## 2. Materials and Methods

### 2.1. Design and Participants

**Figure 1:**
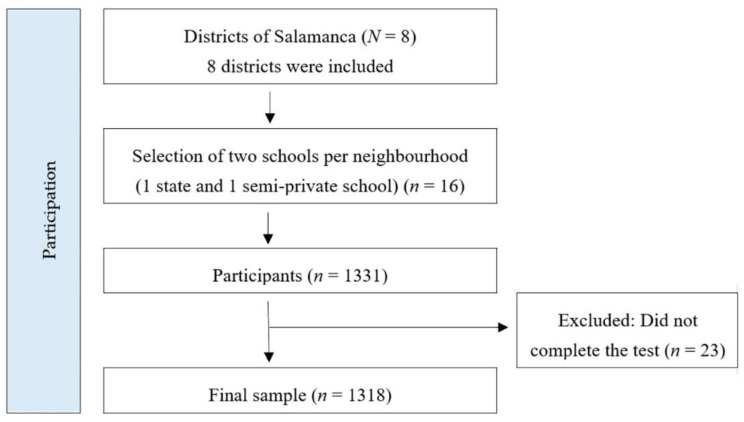
CONSORT chart for sampling selection.

This is a cross-sectional descriptive survey carried out in the city of Salamanca. The sample was taken through stratified multistage random sampling by conglomerates. The 16 participating schools were selected taking into account the social districts as defined by the town council of Salamanca and also the healthcare districts into which the Castilla y Leon Regional Council divides the city. We made sure that each district included a state school and a semi-private school. Amongst the schools that expressed an interest in the research study, 16 were selected for their location in a way that every neighbourhood in the city would be represented. A Consort chart (Figure 1) is included in order to explain the sampling. The sample consisted of 1318 adolescents (50.98% female), between 11 and 18 years of age (14.76 ± 1.61 years old). The total population was 3592, and therefore the range of error was 2.1% (*p* = *q* = 5; *Z* = 2).

The description of the sample, according to the level of practice of physical activity, reveals that 64.5% were classified as active (adolescents who feel active exercised at least 60 min a day), whereas 35.5% were sedentary (the remaining); the BMI reveals that 9% were underweight, 76.9% were within the normal weight range, 12.7% were overweight and 1.4% were obese, a lower percentage than that found at the national level because in this study adolescents were between 11 and 18 years old, while national data collects data on children between 5 and 19 years old. With regards to weight loss diet adherence, 65.3% did not follow a specific weight loss diet, whilst 34.7% did.

### 2.2. Instruments

*Socio-demographic questionnaire*, which compiled information on the sex of the respondents (male and female) as well as age.

*Questionnaire on eating habits*. The international survey Health Behaviour in School-Aged Children (HBSC) questionnaire [48], sponsored by the WHO, was used [49] which contains questions about having a full breakfast and how frequently during the week they consumed certain basic foods. This questionnaire about dietary habits has two dimensions that are interpreted in the following way:

(a) Full breakfast: when in addition to a glass of milk or a piece of fruit, the respondent eats something else. The days per week when the respondent had a full breakfast were quantified (0 to 7 days). Following the recommendations [26] for having a full breakfast, the result “good” was allocated when a full breakfast was eaten every day of the week, “medium” when it was eaten 6 days a week and “poor” when it was eaten five days a week or less. These considerations were based on research studies dealing with the food consumed during childhood and adolescence [25,26,27,50] which state the importance of nutrients and energy for individuals to deal with a long school day.
(b) Weekly consumption frequency by food type. Food was classified as basic (fruit, vegetables, meat and fish) and not very healthy (snacks, soft drinks or sweetened drinks and confectionery). The times a week that these foods were consumed was quantified. The answer options were placed on a scale from 1 to 7: never (1), less than once a week (2), once a week (3), 2–4 days a week (4), 5–6 days a week (5), once a day, every day (6) and every day more than once (7).

*Questionnaire on weight loss diet adherence.* The following question was asked “Are you following a weight loss diet or doing anything to lose weight at present?” There were 4 answer options: (1) no, I think I am at my ideal weight; (2) no, but I should lose some weight; (3) no, because I need to gain weight and (4) yes.

*Questionnaire on the practice of physical activity.* In order to analyse the level of practice of PA, we used the Moderate to Vigorous Physical Activity (MVP) questionnaire [51], consisting of two items which report the number of days a week that the individuals practice at least 60 min of moderate to vigorous PA, both in the previous week and in an average week. The answer scale for both was the same (0 = none, 1 = once, 2 = twice, 3 = three days, 4 = four days, 5 = five days, 6 = six days, and 7 = seven days). Following the recommendations of the World Health Organisation [52], adolescents who exercised at least 60 min a day were considered to be active and the remaining were considered to have a sedentary lifestyle.

Body composition was analysed through the BMI (kg/m^2^ ratio). Weight and height measurements were self-reported by the participants themselves and BMI was calculated. The validity of self-reported data in obtaining BMI in studies with children and youth has already been supported in previous research [20]. BMI was adjusted according to sex and age, using the international reference tables for underweight, normal weight range, overweight and obesity [49,50].

The study strictly complied with all the ethical criteria contained in the Helsinki declaration in its amendment of 2013 for this type of research. The study was approved by the Ethics Committee of Pontifical University of Salamanca (20/07/2020).

Before beginning to collect the data, the 16 participating school centres were contacted for approval and consent of the parents or legal custodians of the students. They were informed of the type of survey proposed and assured them that participation was absolutely voluntary. The students who took part in this survey did not receive financial or academic compensation whatsoever. In the same way, the anonymity and confidentiality of the participants and their data were assured.

At the data collection stage, all the questionnaires were distributed during school time. Four interviewers were trained by one main researcher and they were put in charge of distributing the various questionnaires with the various instruments used.

The participation requirements were voluntary participation, signed authorisation by the parents or legal custodian, and reporting on whether the student was following any kind of nutritional plan or weight loss diet at the time of the study, or during the previous 6 months. Thus, the exclusion criteria were providing incorrect or incomplete answers to any of the items making up the various questionnaires, failure to provide the authorisation by the parents or legal custodian and showing some type of condition during the study (have an injury, be in a cast, remain at rest due to operation, etc.) that might prevent the habitual practice of moderate to vigorous PA. In these cases, participants with these situations did not participate in the survey. No medical evaluation of any student was required.

### 2.3. Data Analysis

The data were analysed using the SPSS version 24.0 statistical piece of software. For the purposes of the statistical study, descriptors, chi-square, Bonferroni post hoc test and the ANOVA analysis as well linear regression were used, taking breakfast and the type of food consumed as dependent variables; and BMI, weigh loss diet and the practice of PA as independent variables; and finally, the co-variables were sex and age.

## 3. Results

### 3.1. Descriptors of the Quality of Breakfast, the Daily Healthy Food Diet and the Occasional Healthy Food Diet

Table 1 analyses the quality of breakfast and the food diet according to sex, age, weight loss diet adherence, BMI and practice of PA.

As Table 1 shows, the quality of breakfast reveals significant differences in all the variables under analysis. For example, boys tend to have a good breakfast (in terms of frequency and consistency) to a greater extent than girls (75.9% against 66.2%); having a poor breakfast (skipping it two or more days a week) is more frequent as adolescents are older (14.7% at 11–12 years of age against 38.6% at 17–18 years of age); students who have a good breakfast (every day and consisting of something more than just a glass of milk) are to a greater extent those who do not follow a weight loss diet (76.2% against 61.1%); breakfast quality worsens as the BMI of the adolescents increases (16.8% are underweight against 29.4% who are obese); practice of PA is a conditioning factor for active students to have a good breakfast in a higher percentage than sedentary students (75.6% against 68.6%).

Analysing the quality of the daily diet, sex, age and practice of PA bring about significant differences. In fact, females have a better and healthier diet in comparison to males (24.7% against 22%) and the diet is better amongst adolescents aged 15–16 (26.2%) compared with adolescents aged 11–12 (19.1%); on the other hand, sedentary adolescents (53.5%) have a poor daily diet compared to the more active adolescents (46%).

With regards to the healthy consumption of the food types that should be consumed only occasionally, significant differences are only found by age. Specifically, its consumption improves with age, thus older adolescents (14.5% at 17–18 years of age) have extensively better diets than the others.

### 3.2. Comparison of Averages (ANOVA) for the Frequency of Consumption of Daily Breakfast on Weekends, Weekdays and during the Entire Week

**Figure 2:**
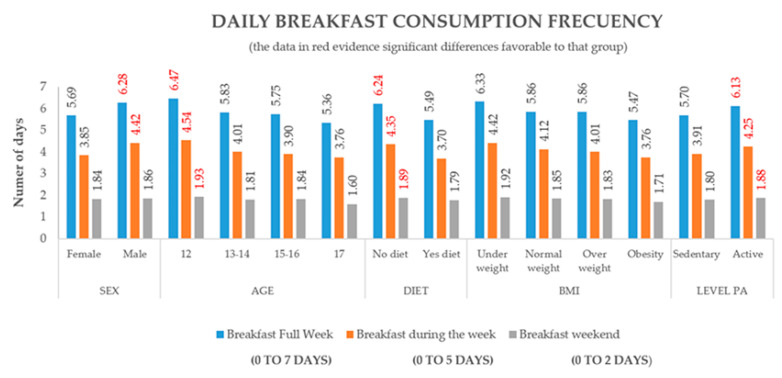
Frequency of breakfast consumption during the entire week, on weekdays and on weekends by sex, age, weight loss diet adherence, body mass index (BMI) and level of physical activity (PA). A dot represents the group with the highest and most significant difference.

Figure 2 analyses the daily consumption frequency of breakfast, with significant differences becoming evident on the weekends (*p* < 0.05), weekdays (*p* < 0.01) and during the entire week (*p* < 0.01). There is a significantly more frequent daily consumption of breakfast over the three analysis points in adolescents between 11 and 12 years of age, those who do not follow a weight loss diet and those who are physically active. Significant differences were found by sex, which pointed at boys showing better results during weekdays and during the entire week. On the other hand, the BMI does not show significant differences.

### 3.3. Comparison of Averages in the Weekly Frequency for the Consumption of the Various Food Types, both Daily Recommended or Occasionally Recommended

Table 2 analyses the frequency of consumption of food types according to sex, age, weight loss diet, BMI and PA. 

On examination of the findings by sex, females reveal a significantly higher consumption of vegetables (*p* < 0.05; 4.5 against 4.3) and confectionery (*p* < 0.01; 3.9 against 3.7) than males; however, males consume more soft drinks (*p* < 0.001; 4.0 against 3.5).

Adherence to a weight loss diet reveals significant differences, with those who do not follow a weight loss diet consuming more vegetables (*p* < 0.05; 4.7 against 4.4), confectionery (*p* < 0.001; 3.9 against 3.3) and nuts (*p* < 0.001; 3.6 against 3.1) than those who follow one.

The level of practice of PA determines that active adolescents compared to sedentary students consume bread and cereal (*p* < 0.001; 5.1 against 5.3), fruit (*p* < 0.001; 5.0 against 4.7), vegetables (*p* < 0.01; 4.5 against 4.3), fish and seafood (*p* < 0.001; 4.0 against 3.7) and soft drinks (*p* < 0.001; 3.9 against 3.5) more frequently.

Age reveals significant differences in the consumption of dairy products with 15–16 year old students consuming more than 17–18 years old students (*p* < 0.01; 6.1 against 5.7); fruit, with 11–12 year old students consuming more than 17–18 year old students in particular (*p* < 0.05; 5.0 against 4.7); confectionery is more consumed by 15–16 year old students compared to 11–12 year old students (*p* < 0.01; 3.9 against 3.6); and soft drinks, with 17–18 year old students consuming them more frequently than 11–12 year old students (*p* < 0.01; 4.1 against 3.5).

The BMI reveals significant differences with greater consumption of fruit in students with normal weight compared to the overweight students in particular (*p* < 0.05; 5.0 against 4.6) greater consumption of bread amongst the obese students compared to overweight students (*p* < 0.05; 5.3 against 5.0) and greater consumption of confectionery in underweight students when compared to obese students (*p* < 0.001; 4.3 against 3.5).

### 3.4. Linear Regression between Quality of Breakfast, Daily Healthy Diet, Foods the Consumption of Which Should Be Only Occasional and the Remaining Variables under Analysis

A linear regression analysis was applied using the Enter method. Quality of breakfast, daily healthy diet and the healthy consumption of foods that should only be consumed occasionally were introduced as dependent variables; weight loss diet, BMI and practice of PA as independent variables; sex and age acted as co-variables (Table 3).

The One-Way ANOVA test shows significant influence on the quality of breakfast (F = 13.230; *p* < 0.001). Specifically, a significant influence in terms of sex (B = 0.154; standard error = 0.049; *t* = 3.129; *p* = 0.002), with boys having a better quality breakfast than girls; age also has a significant impact, in fact, the quality of breakfast decreases as age increases in adolescents (B = −0.167; standard error = 0.059; *t*= −2.822; *p* = 0.005). In the same way, the adherence to a weight loss diet has a significant impact, with the individuals following a weight loss diet eating a lesser-quality breakfast (B = −0.194; standard error = 0.056; *t*= −3.490; *p* < 0.001). The BMI also has significant influence (B = −0.019; standard error = 0.009; *t*= −2.137; *p* < 0.05), associating a better-quality breakfast with lower BMI values.

## 4. Discussion

The findings of our study reveal significant associations between dietary habits, breakfast habits and the other variables under analysis (sex, age, weight loss diet, BMI and physical activity).

In line with the research and the national data [15], boys have a better quality breakfast than girls, and this habit worsens with age. Following a weight loss diet impacts on the quality of breakfast by reducing it according to this study: restrictions in breakfast ingredients or skipping breakfast is more frequent amongst girls and at older ages, in line with similar studies [44]. Such differences can derive from the fact that girls tend to be more worried about their appearance and skip breakfast in order to control their weight [53], an unhealthy habit in childhood and adolescence [25]. BMI is lower in adolescents eating a quality breakfast, in the same way as in other similar studies [40,42,53]. Lastly, adolescents who practice PA have a better quality breakfast, which is also similar to other research studies [54,55], with such active adolescents also appearing to be more concerned with keeping a balanced diet.

With regard to the quality of daily diet, sex and age are major determining factors, with quality being better amongst girls and at older ages in both sexes, following the trend of the national data [15] in recent research studies [56,57]. Active adolescents eat better, following the line of other research studies [58], supporting the idea that healthy habits tend to happen together [59].

With regards to frequency, our research showed that the younger they are, the better the weekly breakfast habits are, in line with the national statistics. Not following a weight loss diet and being an active adolescent was also associated with a good frequency of breakfast. In terms of the daily or occasional consumption of certain food types, age is a variable to consider: the older the adolescent, the better the consumption of dairy products and the worse the consumption of fruit and vegetables, confectionery and soft drinks, in line with the national research study [15]. Following a weight loss diet has an impact on the frequency with which the various food types are eaten; thus, adolescents on a weight loss diet limit the number of nuts they eat. In terms of food intake, BMI is also relevant, with this value being lower when fruit and vegetables are eaten daily and higher when confectionery and soft drinks are consumed frequently, in line with other recent research [60]. Adolescents practicing PA eat more bread and cereal, fruit and vegetables, fish and soft drinks, which are food products that provide more energy and nutrients.

The regression analysis allows us to consider which variables can better predict a quality breakfast and a healthy diet, with this research study revealing that sex and age are determining factors for a quality breakfast (which is better in males and in younger adolescents of both sexes). Our analysis also allowed us to establish that during this stage of life, following a weight loss diet has an impact on the quality of breakfast, making it worse, as other research studies have revealed [61], and that a better quality breakfast can be associated with lower BMI values [40,41]. In terms of food intake, the regression analysis did not allow us to establish evidence, potentially because of the impact of other psychosocial factors on the consumption of food types (family habits, use of coffee bars or school canteens, the media, etc.).

These findings appear to be relevant, particularly because the medical history of the majority of patients with eating disorders tend to include unsupervised weight loss diets. If we take into account that the most frequent pathologies (compulsive eating disorder, anorexia and bulimia nervosa) are associated with inadequate consumption of meals, which are either excessive or insufficient, we can certainly arrive at an explanation for the origin of any eating habits that become evident in university students [62] and are created in previous stages of life. Thus, dangerous eating habits are associated with the percentage of body fat in adolescents. In the research study carried out by Sánchez-Zamorano [63] with a cohort of over two thousand adolescents for a term of three years, the multiple linear regression models used with mixed effects lead to the conclusion that following an unsupervised weight loss diet can be associated with an increase in the percentage of body fat, which causes effects that are the opposite to what was intended. It is important to highlight the relevant role that family relationships play in this respect. It is necessary for the family to become involved, as well as having school programs with differentiated instruction designs between sexes.

With regards to personality patterns, individuals who have previously followed or currently follow dangerous weight loss diets tend to also be those with the highest scores in variables such as neuroticism, obsession with thinness, bulimia, body dissatisfaction and asceticism. They also reveal traits of perfectionism, emotional negativity, symptoms of depression, low self-esteem and negative impulsiveness [64]. In terms of the aforementioned personality traits (perfectionism, obsession with thinness, etc.) it is very important to determine how these have shaped the identity of the adolescent. Considering the whole emotional and experiential universe of adolescents (family bonds, peer relationships) can help change their behaviour (following weight loss diets) and redirect their life projects [65]. Apart from the individual sphere, other psychosocial factors (exposure to the social ideal of thinness in girls, the internalisation of that thinness ideal or the expectations to become thin) must be considered as they could predict an increase in the levels of body dissatisfaction. On this account, intervention programs must include the modification of inadequate cognitive patterns in order to eliminate dangerous behaviours [66].

Some of the strengths of this research study are associated with the volume and the type of sample, the use of validated measurement instruments which are specific for this population group and research type, as well as the relationship existing between breakfast and a healthy diet with variables that indicate healthy habits such as BMI, diet type and PA practice frequency.

As a limitation to this study, we can mention that the design does not help draw cause relationships between the various variables under analysis. In future research projects we would suggest that social status be included as a cofounding variable between the quality of the diet and the other variables under study.

## 5. Conclusions

In conclusion, the findings of this research study reveal that sex and age are relevant for the quality of breakfast and the consumption of the various food groups. Additionally, following a weight loss diet leads to a lesser quality and frequency of breakfast intake, and this habit, together with an adequate consumption of the food groups, is better in adolescents with lower BMI and more active adolescents.

This study provides scientific evidence on the impact that variables of sex, age, weight loss diet, BMI and PA have on the consumption of a quality breakfast and the adequate consumption of the various food groups. It is important to consider all of them as interrelated in the promotion of healthy habits amongst the adolescent population. In this sense, recent research [67] reveals the relevance of promoting good dietary habits from all the areas of development of the adolescent, i.e., family, school and community on account of the consequences that this has for their health in the short and long term. Current research studies [63,68] into different contexts point out the important role that this early nutritional education has in the emergence of eating disorders in adolescents, as well as the need to draw up strategies and protocols of action once these appear. What is most relevant from the point of view of this scenario is that the findings deriving from this study enable us to contribute to the completion of studies on personality patterns and other psychologic variables, which are the result of habits learned in development stages previous to adulthood, and the chronification of which is highly resilient to intervention [61].

One of the main contributions of this research study is based on the relevance of adopting healthy habits for the improvement of people’s health [57,69] and the importance that this line of research has from an educational perspective within a Spanish and European context [37,70]. This research study can contribute up-to-date information that allows to draft strategies for awareness and promotion of the improvement and consolidation of healthy habits which require the cooperation of the family, the school, the social agents and the media [55,71].

## Figures and Tables

**Table 1:** Comparison of quality of breakfast and type of healthy diet by sex, age, weight loss diet adherence, BMI and practice of PA.

			Quality of Breakfast ^1^				Quality of Healthy Diet Daily Consumption ^2^				Quality of Healthy Diet Occasional Consumption ^3^			
Variable	Classification		Poor (*n* = 323)	Medium (*n* = 60)	Good (*n* = 935)	*P* Value ^4^	Poor (*n* = 672)	Medium (*n* = 494)	Good (*n* = 152)	*P* Value ^4^	Poor (*n* = 801)	Medium (*n* = 401)	Good (*n* = 116)	*P* Value ^4^
**Sex**	Man	%	19.5	4.6	75.9	<0.001 ***	49.4	28.6	22.0	0.47	63.8	28.6	7.6	0.069
	Woman	%	29.3	4.5	66.2		52.5	22.8	24.7		57.9	32.1	3.7	
**Age**	11–12 y.o.	%	14.7	3.5	81.8	<0.001 ***	51.2	29.7	19.1	0.018	55.5	36.4	8.1	0.029 *
	13–14 y.o.	%	29.2	5.7	65.1		55.5	19.9	24.6		62.3	28.8	8.9	
	15–16 y.o.	%	28.1	4.3	67.6		47.5	26.4	26.2		64.1	27.3	8.5	
	17–18 y.o.	%	38.6	6.0	55.4		55.4	20.5	24.1		60.2	25.3	14.5	
**Weight Loss Diet Adherence**	No	%	19.6	4.2	76.2	<0.001 ***	49.8	26.3	23.9	0.462	62.3	29.8	7.9	0.149
	Yes	%	33.7	5.3	61.1		53.4	24.1	22.5		57.5	31.9	10.5	
**BMI**	Underweight	%	16.8	6.7	76.5	<0.001 ***	52.9	19.3	27.7	0.071	63.9	27.7	8.4	0.604
	Normal weight	%	24.9	3.8	71.3		50.7	25.9	23.4		60.8	30.6	8.6	
	Overweight	%	27.2	7.1	65.7		52.4	29.8	17.9		59.5	29.8	10.7	
	Obesity	%	29.4	5.9	64.7		35.3	17.6	47.1		52.9	47.1	0.0	
**Practice of PA**	Sedentary	%	26.0	5.3	68.6	0.017 *	53.5	23.6	23.0	0.019	59.1	31.9	8.9	0.194
	Active	%	21.4	3.0	75.6		46.0	29.9	24.1		64.1	27.4	8.5	

^1^. Answer scale: Poor (when breakfast was consumed 5 days a week or less frequently); Medium (when breakfast was consumed 6 days a week); Good (when breakfast was consumed every day of the week). ^2^. Answer scale: Poor (has 1–2 healthy eating habits a day); Medium (has 3–4 healthy eating habits a day); Good (has 5–7 healthy eating habits a day); ^3^. Answer scale: Poor (does not follow the recommendations as to consumption of confectionery and soft drinks); Medium (does not follow the recommendations as to consumption of confectionery or soft drinks); Good (does follow the recommendations as to consumption of confectionery and soft drinks). * *p* < 0.01; *** *p* < 0.001. ^4^. Chi-square.

**Table 2:** Frequency of consumption of the various types of food, both daily recommended and occasionally recommended according to sex, diet adherence, level of PA, age and BMI.

	% (n)	Dairy Products	Bread, Cereals	Fruit	Lean Meats	Vegetables	Fish and Seafood	Sweet	Soft Drinks	Nuts
Factors		*M (SD)*	*M (SD)*	*M (SD)*	*M (SD)*	*M (SD)*	*M (SD)*	*M (SD)*	*M (SD)*	*M (SD)*
Sex										
Male	50.1	6.0 (1.2)	5.2 (1.5)	4.8 (1.6)	4.8 (1.1)	4.3 (1.3)	3.9 (1.3)	3.7 (1.5)	4.0 (1.7)	3.6 (1.1)
Female	49.9	5.9 (1.4)	5.3 (1.4)	4.9 (1.6)	4.7 (1.0)	4.5 (1.3)	3.8 (1.1)	3.9 (1.5)	3.5 (1.6)	3.5 (1.2)
Total		5.9 (1.3)	5.2 (1.4)	4.9 (1.6)	4.7 (1.1)	4.4 (1.3)	3.8 (1.2)	3.8 (1.5)	3.8 (1.7)	3.5 (1.2)
*p ^a^*		0.053	0.712	0.281	0.223	0.016 *	0.008 *	0.008 **	<0.001 ***	0.350
Diet										
Yes	11.9	5.8 (1.5)	5.1(1.6)	5.0 (1.6)	4.7 (1.1)	4.7 (1.4)	3.8 (1.3)	3.3 (1.5)	3.7 (1.6)	3.1 (1.2)
No	88.1	6.0 (1.3)	5.3 (1.4)	4.9 (1.6)	4.7 (1.0)	4.4 (1.3)	3.8 (1.1)	3.9 (1.5)	3.8 (1.7)	3.6 (1.2)
*p* ^a^		0.129	0.184	0.203	0.559	0.016 *	0.699	<0.001 ***	0.518	<0.001 ***
Physical Activity										
Sedentary	35.5	5.9 (1.3)	5.1 (1.4)	4.7 (1.7)	4.7 (1.0)	4.3 (1.3)	3.7 (1.1)	3.8 (1.5)	3.5 (1.7)	3.5 (1.1)
Active	64.5	6.0 (1.3)	5.3 (1.4)	5.0 (1.6)	4.8 (1.1)	4.5 (1.3)	4.0 (1.2)	3.8 (1.5)	3.9 (1.7)	3.6 (1.2)
*p* ^a^		0.192	0.001 **	<0.001 ***	0.116	0.005 **	<0.001 **	0.540	<0.001 ***	0.263
Age										
11–12 (a)	33.0	6.0 (1.3)	5.3 (1.4)	5.0 (1.5)	4.7 (1.0)	4.5 (1.2)	3.9 (1.1)	3.6 (1.4)	3.5 (1.6)	3.4 (1.2)
13–14 (b)	21.4	5.8 (1.4)	5.2 (1.5)	4.7 (1.7)	4.7 (1.1)	4.4 (1.4)	3.8 (1.4)	3.9 (1.7)	4.1 (1.8)	3.5 (1.2)
15–16 (c)	39.3	6.1 (1.2)	5.3 (1.4)	4.9 (1.6)	4.8 (1.0)	4.5 (1.2)	3.8 (1.0)	3.9 (1.4)	3.7 (1.6)	3.5 (1.1)
17–18 (d)	6.3	5.7 (1.4)	5.1 (1.3)	4.6 (1.7)	4.7 (1.1)	4.4 (1.3)	3.8 (1.2)	3.9 (1.7)	4.1(1.8)	3.7 (1.4)
*p* ^b^		0.002 **	0.632	0.008 **	0.213	0.734	0.460	0.002 **	<0.001 ***	0.252
Multiple comparision ^c^		b = d < c *		a * > b = d				a < b * a < c **	a ** < b = d b > c **	
BMI										
Underweight a)	9.0	6.0 (1.3)	5.3 (1.5)	4.8 (1.6)	4.8 (1.2)	4.5 (1.4)	3.8 (1.3)	4.3(1.8))	3.7 (1.6)	3.6 (1.4)
Normal weigh (b)	76.9	6.0 (1.3)	5.3 (1.4)	5.0 (1.6)	4.8 (1.0)	4.5 (1.3)	3.9 (1.2)	3.8 (1.5)	3.7 (1.7)	3.5 (1.1)
Overweight (c)	12.7	5.8 (1.3)	5.0 (1.4)	4.6 (1.7)	4.7 (1.0)	4.3 (1.2)	3.8 (1.2)	3.5 (1.4)	3.9 (1.6)	3.4 (1.2)
Obesity (d)	1.4	5.8 (1.1)	5.3 (1.2)	4.7 (1.9)	4.2 (1.5)	4.8 (1.5)	3.2 (1.1)	3.3 (1.1	4.3 (1.8)	3.5 (1.5)
*p* ^b^		0.491	0.041 *	0.025 *	0.114	0.322	0.057	<0.001 ***	0.388	0.317
Multiple comparision ^c^			b > c *	b > c *				a > b ** a > c *** a > d * b ** > c = d		

Note: a: Student´s t; b: ANOVA; c: Bonferroni; * *p* < 0.05; ** *p* < 0.01; *** *p* < 0.001.

**Table 3:** Regression analysis: quality of breakfast, occasional healthy diet and daily healthy diet (dependent variables) and weight loss diet adherence, body mass index and level of practice of physical activity (independent variables), adjusted to co-variables of sex and age.

		Unstandardised Coefficients		Standardised Coefficients			OneWay ANOVA			
		*B*	Standard Error	*B*	t	*p*	Sum of Squares RG(RS)	*MS* RG(RS)	*F*	*p*
BREAKFAST	Cons.	1.798	0.180	-	10.013	<0.001	46.716 (918.093)	9.343 (0.706)	13.230	<0.001
	Sex	0.154	0.049	0.090	3.129	0.002				
	Age	−0.167	0.059	−0.079	−2.822	0.005				
	WL Diet	−0.194	0.056	−0.107	−3.490	<0.001				
	BMI	−0.019	0.009	−0.067	−2.137	0.033				
	PA	0.017	0.013	0.040	1.383	0.167				
		*R*: 0.220 - *R*^2^: 0.048 - Adjusted *R*^2^: 0.045 - *df*: 5. 1305 - Durbin-Watson: 0.89								
DAILY DIET	Cons.	0.257	0.146	-	1.762	0.078	3.726 (607.980)	0.745 (0.468)	1.593	0.159
	Sex	0.008	0.040	0.006	0.208	0.835				
	Age	−0.041	0.048	−0.024	−0.844	0.399				
	WL Diet	−0.075	0.045	−0.052	−1.662	0.097				
	BMI	−0.016	0.007	0.072	−2.237	0.025				
	PA	0.012	0.10	0.034	1.171	0.242				
		*R*: 0.078 - K^2^: 0.006 - Adjusted *R*^2^: 0.002 - *df*:5. 1305 - Durbin-Watson: 0.013								
OCCASIONAL DIET	Cons.	0.508	0.139	-	3.641	<0.001	4.596 (553.425)	0.919 (0.426)	2.159	0.056
	Sex	−0.068	0.038	−0.052	−1.785	0.074				
	Age	−0.064	0.046	−0.040	−1.382	0.167				
	Wl Diet	0.060	0.043	0.044	1.398	0.162				
	BMI	0.002	0.007	0.008	0.262	0.793				
	PA	−0.009	0.010	−0.026	−0.870	0.384				
		*R*: 0.091 - *R*^2^: 0.008 - Adjusted *R*^2^: 0.004 - *df*: 5. 1305 - Durbin-Watson: 0.190								

Cons.: constant term; Sex (0 = girl and 1 = boy); Age: continuous value (12 to 16); Diet: no weigh loss diet adherence (0) and weight loss diet adherence (1); BMI: body mass index (0 = underweight, 1 = normal weight, 2 = overweight and 3 = obesity); PA: physical activity (0 = sedentary and 1 = active); RG: Regression; RS: Residual.
